# Characterization of *Bacillus velezensis* TJS119 and its biocontrol potential against insect pathogens

**DOI:** 10.3389/fmicb.2024.1361961

**Published:** 2024-05-09

**Authors:** Kook-Il Han, Young Ho Nam, Byung Su Hwang, Jeong Tae Kim, Jum Oc Jung, Eunsun Kim, Mi-Hwa Lee

**Affiliations:** ^1^Using Technology Development Department, Nakdonggang National Institute of Biological Resources (NNIBR), Sangju, Republic of Korea; ^2^Industrial Insect and Sericulture Division, National Institute of Agricultural Sciences, Rural Development Administration (RDA), Wanju, Republic of Korea

**Keywords:** *Bacillus velezensis*, *Protaetia brevitarsis seulensis larvae*, biosynthetic genes, *Metarhizium anisopliae*, insect disease, green muscardine, biocontrol

## Abstract

**Introduction:**

The white-spotted flower chafer (*Protaetia brevitarsis seulensis*), which is widely distributed in Asian countries, is traditionally used in oriental medicine. However, its larvae are prone to severe damage by green muscardine disease (caused by *Metarhizium anisopliae*) during breeding. The aim of this study was to characterize *Bacillus velezensis* TJS119, which has been isolated from freshwater, and investigate its potential as a biocontrol agent against *M. anisopliae* in insects.

**Methods:**

TJS119 was obtained from freshwater samples in the Republic of Korea and was classified as *B. velezensis*. We evaluated its *in vitro* antifungal effect, sequenced the bacterial whole genome, mined genes responsible for the synthesis of secondary metabolites, performed secondary metabolite analysis Ultra performance liquid chromatography-mass spectrometry (UPLC–MS/MS), and conducted bioassays for determining green muscardine disease control ability.

**Results:**

*Bacillus velezensis* TJS119 inhibited the mycelial growth of *M*. *anisopliae in vitro*. The size of the *B. velezensis* TJS119 genome was estimated to be 3,890,913 bp with a GC content of 46.67% and 3,750 coding sequences. Biosynthetic gene clusters for secondary metabolites with antifungal activity were identified in the genome. Lipopeptides, including fengycin secreted by TJS119 exhibit antifungal activity. Application of TJS119 for the biocontrol against green muscardine disease increased the viability of white-spotted flower chafer by 94.7% compared to the control.

**Discussion:**

These results indicate that *B. velezensis* TJS119 is a potential biocontrol agent for insect pathogens.

## 1 Introduction

The white-spotted flower chafer *Protaetia brevitarsis seulensis* is traditionally used in oriental medicine. It is widely distributed in Asian countries including Korea, Southeast Asia, and China (Pan J. et al., 2017). Belonging to the family Scarabaeidae, which is globally distributed with nearly 6,200 species and 267 genera (Suh and Kang, 2012), white-spotted flower chafers play an important ecological role. The larvae of white-spotted flower chafers inhabit soil and feed on decaying organic matter. Thus, they are exposed to a wide range of pathogenic microbes that attack their immune systems, thereby experiencing considerable selective pressure (Li et al., 2019). In traditional Korean medicine, the larvae are used to treat microbial infections. Additionally, the white-spotted flower chafer is widely marketed as an edible insect, generating high revenue. In insect farms, the larvae are subjected to mass mortality owing to the occurrence of green muscardine disease.

Green muscardine infection is caused by a fungus belonging to the genus *Metarhizium*. The name of the disease is based on the observation that after the death of the host insect, the fungus covers its cuticle with a layer of green spores. Insect mycologists and microbial control specialists use the name “green muscardine” to specifically refer to infections caused by *Metarhizium* spp., with many of them infecting a wide range of insects, including the larvae of *P. brevitarsis seulensis.* Green muscardine reportedly affects over 200 known insect species (Calderone, 2002).

Microorganisms with antifungal activity are widely used in agriculture. Among these, *Bacillus* spp. have demonstrated high effectiveness as biocontrol agents. They produce spores and several bioactive compounds, making them resilient against challenging environmental conditions. Therefore, *Bacillus* spp. are among of the most extensively studied bacteria in the field of biocontrol (Li et al., 2021; Pang et al., 2021). *Bacillus subtilis*, *B. amyloliquefaciens*, *B. cereus*, *B. megaterium*, and *B. velezensis* are the most commonly used biocontrol agents, because they produce phytohormones that promote plant growth and a wide range of secondary metabolites that suppress competitive plant pathogens (Lugtenberg and Kamilova, 2009). Recently, *B. velezensis* has been reclassified as a heterotypic synonym of *B. amyloliquefaciens* subsp. *plantarum*, *B. methylogrophicus*, and *B. oryzicola* (Dunlap et al., 2015). Several strains of *B. velezensis* have been extensively used in agriculture and biotechnology due to their environmental safety, ease of industrial production, and biocontrol efficacy (Rabbee et al., 2023). These include *B. velezensis* BR-1 (Zhou et al., 2022), HNA3 (Zaid et al., 2022), GH1-13 (Kim et al., 2017b), S3-1 (Jin et al., 2017), M75 (Kim et al., 2017a), LS69 (Liu et al., 2017), 9912D (Pan H. Q. et al., 2017), and S499 (Molinatto et al., 2016). However, the relative efficacy of *B. velezensis* TJS119 against insect pathogens and its mechanism of biocontrol remain unknown.

The aim of this study was to investigate the mechanisms underlying the biocontrol ability of *B. velezensis* TJS119 and to determine its genome sequence. The results of bioassays indicated that *B. velezensis* TJS119 is a potential biocontrol agent that can be used against green muscardine (*M. anisopliae*). Genomic and secondary metabolite analysis were performed to determine the mechanism by which *B. velezensis* TJS119 controls diseases. Overall, the results of the present study support the application of *B. velezensis* for disease control in *Protaetia brevitarsis seulensis* larvae.

## 2 Materials and methods

### 2.1 Isolation of bacterial strains

One hundred and eighty-six bacterial strains were isolated from freshwater in the Nakdong River (128.26°E, 36.43°N, June 2021), Sangju, Republic of Korea. Freshwater samples were serially diluted in sterilized distilled water and inoculated on tryptic soy agar plates (TSA; BD Biosciences). The plates were incubated at 30°C for 5 days. Single colonies were selected and continuously sub-cultured in fresh TSA to obtain pure cultures.

### 2.2 Screening of biological control bacteria against green muscardine

Each bacterium was activated at 28°C for 24 h with agitation at 200 rpm and subsequently inoculated into tryptic soy broth (TSB) liquid medium with liquid loading of 10 mL·L^−1^ in a 200 mL Erlenmeyer flask under identical conditions for 2 days. Aseptic filtrate of the bacterium was obtained using an aseptically sterilized bacterial filter with a pore size of 0.22 μm. The targeted pathogen, *M. anisopliae* strain KACC 40969, was obtained from the Korean Agricultural Culture Collection (KACC). *Metarhizium anisopliae* was cultured on potato dextrose agar (PDA; BD Biosciences) medium at 28°C for 1 month, and then washed with sterile distilled water to obtain conidia. A hemocytometer was used to adjust the conidial suspension to 1 × 10^6^ conidia/mL. *Metarhizium anisopliae* suspension was prepared by adding 1 × 10^5^ conidia/g to PDA medium. Thereafter, a paper disk (8 mm) was placed on the PDA and loaded with 100 μL of the filtered culture supernatant of each bacterium. The plates were inverted and incubated at 28°C for 3 days. The inhibitory zone was determined in millimeters (mm) using the vernier calipers.

### 2.3 Genome sequencing and analysis of bacterial

For genome sequencing, TJS119 cells were cultured in TSA at 30°C for 48 h, and their genomic DNA (gDNA) was extracted using the Wizard Genomic DNA Kit (Promega, United States) according to the manufacturer’s instructions. The whole genome was sequenced using an Illumina NovaSeq platform at Macrogen (Seoul, Republic of Korea). gDNA libraries were generated using the TruSeq Nano DNA High Throughput Library Prep Kit (Illumina). The generated short and long reads were quality-filtered and adapter-trimmed using the Trimmomatic software (version 0.36) (Bolger et al., 2014). The quality of the Illumina reads was assessed using FastQC (v0.11.5; https://www.bioinformatics.babraham.ac.uk/projects/fastqc) for Illumina outputs. Library sequencing data were assembled using SPAdes version 3.15 (Bankevich et al., 2012). Genome sequence contamination was assessed using the contamination estimator in BUSCO (Simão et al., 2015). Protein-coding sequences, tRNAs, and insertion sequence elements were predicted using Prokka (version 1.14.6) (Seemann, 2014), tRNA-scan-SE (v2.0.9), and ISEScan (v1.7.2.3), respectively. Average nucleotide identity (ANI) values were calculated using OrthoANI^1^ (Lee et al., 2016). The default settings were used for all software analysis unless otherwise indicated. The draft genome sequence of TJS119 has been deposited at GenBank under the accession number JAXQPU000000000. Phylogenetic trees based on 92 bacterial core genes were constructed with an up-to-date bacterial core gene set (UBCG) (Na et al., 2018) using the maximum-likelihood method (Felsenstein, 1981). The whole-genome sequence of TJS119 was processed using the antibacterial version of the antibiotic and secondary metabolite analysis shell (antiSMASH, version 7.0.0) webserver.^2^ Biosynthetic gene clusters (BGCs) were identified by aligning the sequence against different genomes using the BLAST tool of the National Center for Biotechnology Information (NCBI) database.^3^ The identified BGCs were compared with those of other microbes compiled in the Minimum Information for Biosynthetic Gene Clusters (MIBiG) database^4^ to identify whether similar pathways exist in other organisms. Virulence factors were confirmed using the VFanalyzer online tool (Liu et al., 2022) (accessed on 4 March 2024). The default settings were used for all software analysis unless otherwise indicated. The PathogenFinder 1.1 online tool (Cosentino et al., 2013) was employed to conduct bacterial pathogenicity estimation. The assembled fasta files were uploaded to PathogenFinder, and Firmicutes was selected as the organism class (accessed on 4 March 2024).

### 2.4 Extraction of secondary metabolites from TJS119

To confirm the activity of the selected strain, it was inoculated in 1 L of TSB and incubated at 30°C for 3 days with constant shaking in a shaker incubator. Cell-free supernatant was then collected via centrifugation at 7,000 × *g* for 20 min at 4°C, solvent-extracted twice using an equal volume of n-butanol in a separating funnel with vigorous shaking for approximately 5 min, and allowed to stand for 30 min. The aqueous and organic phases were separated, and the organic phase was collected and evaporated using a rotary evaporator under vacuum at 35°C. After evaporation, the dried crude organic extract was dissolved in methanol. The crude extract was subjected to paper disk diffusion to confirm the presence of antifungal compounds. A hemocytometer was used to adjust the concentration conidial suspension to 1 × 10^6^ conidia/mL. *Metarhizium anisopliae* plates ware prepared by adding 1 × 10^5^ conidia/g to PDA medium. To these plates, 50, 100, 200, or 300 μL of the crude extract was added on the disk of each plate, with pure n-butanol as a control. The plates were then incubated at 28°C for 3 days and observed for antifungal activity.

### 2.5 Identification of antifungal compounds using UPLC–MS/MS analysis

The crude extract was passed through a syringe filter (0.22 μm). An Exion UPLC and a SCIRX QTRAP 4500 mass spectrometer equipped with an ESI interface were used (SCIEX, Framingham, MA, United States). The Luna Omega polar C_18_ column (150 mm × 2.1 mm, 1.6 μm; Phenomenex) was employed for chromatographic separation at 35°C. The mobile phase consisted of 0.1% formic acid (A) and acetonitrile (B), at a flow rate of 0.25 mL/min. Gradient elution was performed as follows: 0–1 min, 10% B; 1–16 min, 10–95% B; 16–19 min, 95% B. Nitrogen (N2) was used as the collision gas, the ion spray voltage was 5.5 kV, and the temperature was 600°C. The declustering potential (DP) voltage and collision energy were set at 30 and 10, respectively.

### 2.6 Bioassay

**Figure 1:**
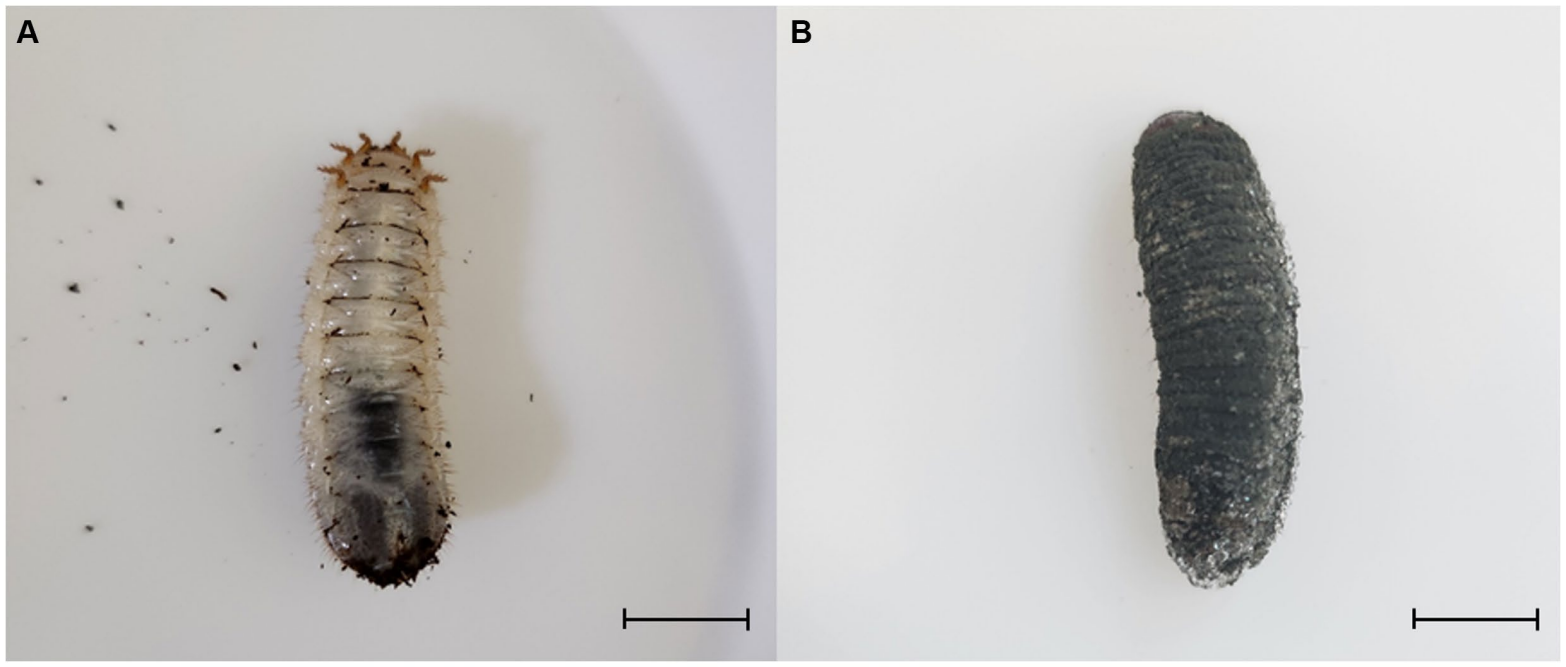
Third instar larvae of *Protaetia brevitarsis* were observed: **(A)** a non-infected larva, and **(B)** a larva infected with *Metarhizium anisopliae*. The infected larvae exhibited stiffness and displayed dark green spores on their epidermis. Scale bar: 10 mm.

*Protaetia brevitarsis* (third instar) larvae were obtained from a local farm (Sangju Gapjangsan white grub farm, Sangju, Republic of Korea). They were reared at 25°C under 65% relative humidity in the dark. Plastic cages (L × W × H = 180.00 cm × 460.00 cm × 160.00 cm) were used for rearing. The experiment was conducted with 100 larvae placed in 2 kg of sterilized sawdust. *Metarhizium anisopliae* was cultured on PDA (BD Biosciences) medium at 28°C for 1 month, and then washed with sterile distilled water to obtain conidia. The conidia suspension was used at a concentration of 1 × 10^5^ conidia/mL per 100 g of sawdust. In the experimental group, *B. velezensis* TJS119 strain was used at a concentration of 1 × 10^7^ CFU/g; water was added to the control group. The final sawdust moisture content was 60%. After treating with the fungus, we confirmed the mortality of larvae due to green muscardine disease for 5 weeks. The presence of green muscardine disease was determined based on the formation of *M. anisopliae* conidia on the epidermis of dead larvae (Figure 1). We calculated the average number of individuals affected by the disease, aggregated through experiments conducted with three repetitions.

$$\mathrm{Dise}ase\;\mathrm{index}\left(\%\right)=\frac{\mathrm{count}\;\mathrm{of}\;\mathrm{diseased}\;\mathrm{larvae}}{\mathrm{total}\;\mathrm{count}\;\mathrm{of}\;\mathrm{tested}\;\mathrm{larvae}}\;x\;100$$

$$\begin{array}{l} \mathrm{Biocontrol}\;\mathrm{effect}\left(\%\right)=\\ 100-\frac{\mathrm{disease}\;\mathrm{incidence}\;\mathrm{rate}\;\mathrm{in}\;\mathrm{experiment}\;\mathrm{group}}{\mathrm{disease}\;\mathrm{incidence}\;\mathrm{rate}\;\mathrm{in}\;\mathrm{control}\;\mathrm{group}}\;x\;100 \end{array}$$

## 3 Results

### 3.1 Isolation and identification of *Bacillus velezensis* TJS119

**Figure 2:**
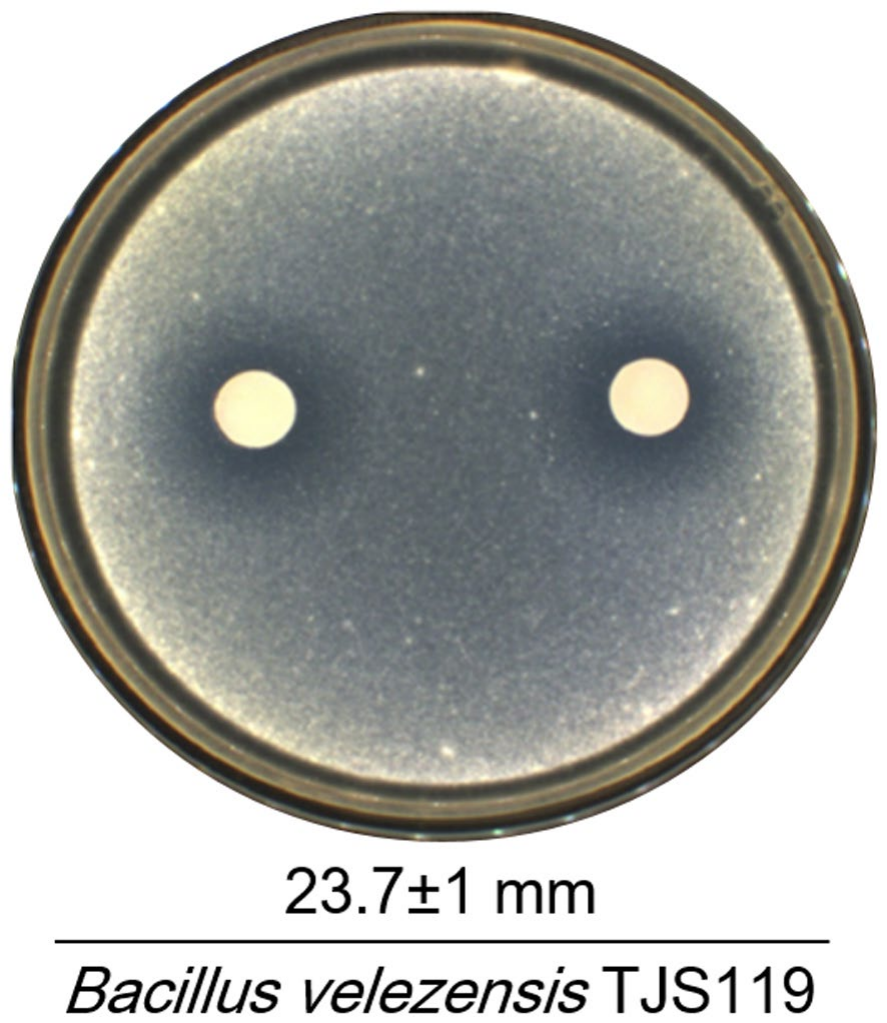
Antagonistic effect of *Bacillus velezensis* TJS119 on *Metarhizium anisopliae*. Values given are mean ± standard deviation of duplicate measurements.

Out of 186 bacterial isolates obtained from the freshwater samples of the Nakdong River, isolate TJS119 exhibited the highest degree of fungal growth inhibition. *Bacillus velezensis* TJS119 was chosen for the maximum antagonistic activity against *M. anisopliae* in culture media, with an antagonistic zone width of 23.7 ± 1 mm (Figure 2). The strain has been maintained at the Nakdonggang National Institute of Biological Resources (NNIBR) and deposited as strain KACC 81272BP at the “Korean Agricultural Culture Collection (KACC)” of Wanju-gun, Korea.

### 3.2 Genome information of *Bacillus velezensis* TJS119

**Figure 3:**
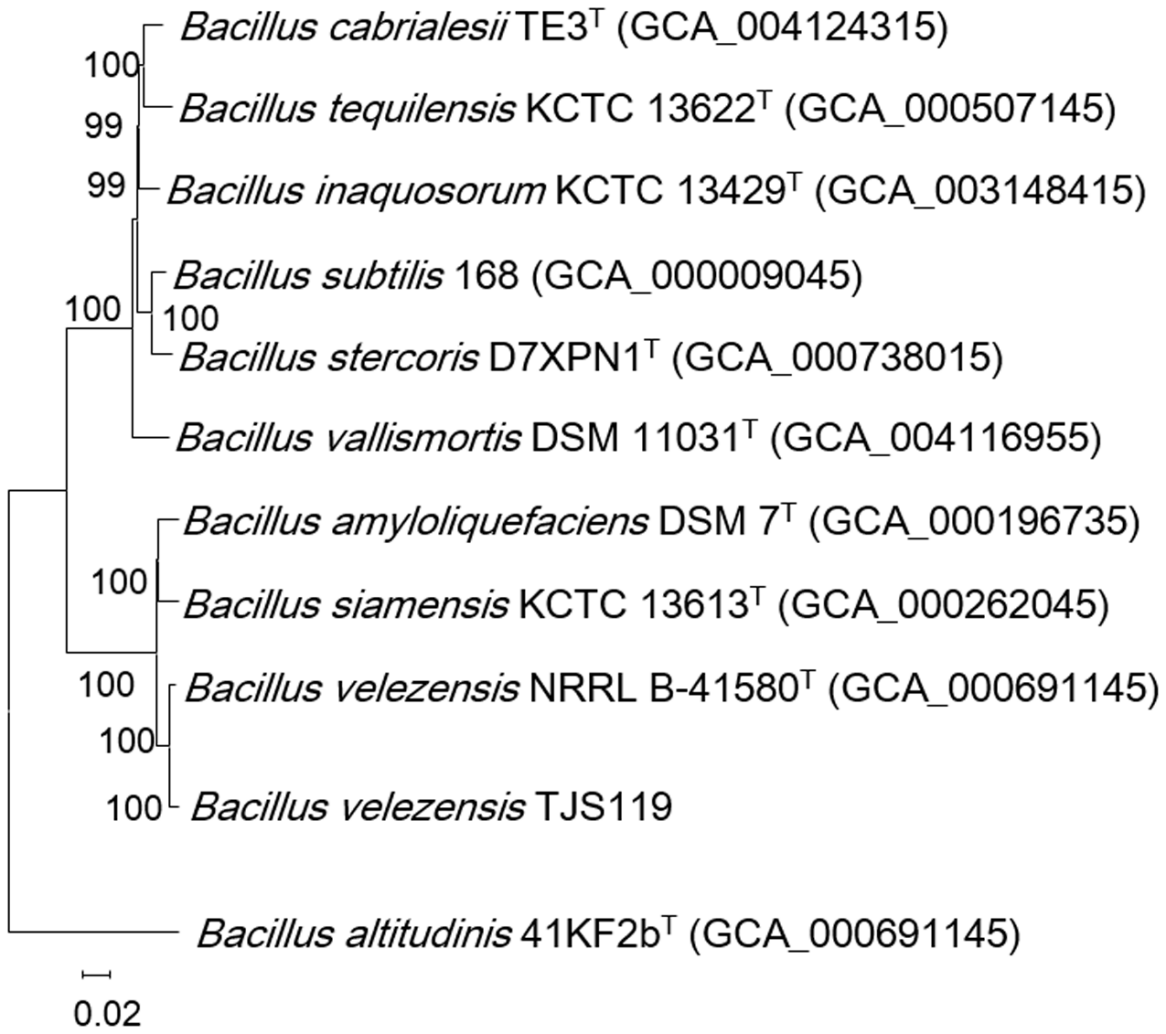
Phylogenomic tree based on 92 bacterial core gene sequences, reconstructed with the maximum-likelihood method, showing the position of TJS119 among the related taxa within the genus *Bacillus*. Genome accession numbers are indicated in parentheses. The numbers at the nodes indicate the gene support index. Bar: 0.02 substitutions per position. The 92 bacterial core genes were *alaS*, *argS*, *aspS*, *cgtA*, *coaE*, *cysS*, *dnaA*, *dnaG*, *dnaX*, *engA*, *ffh*, *fmt*, *frr*, *ftsY*, *gmk*, *hisS*, *ileS*, *infB*, *infC*, *ksgA*, *lepA*, *leuS*, *ligA*, *nusA*, *nusG*, *pgk*, *pheS*, *pheT*, *prfA*, *pyrG*, *recA*, *rbfA*, *rnc*, *rplA*, *rplB*, *rplC*, *rplD*, *rplE*, *rplF*, *rplI*, *rplJ*, *rplK*, *rplL*, *rplM*, *rplN*, *rplO*, *rplP*, *rplQ*, *rplR*, *rplS*, *rplT*, *rplU*, *rplV*, *rplW*, *rplX*, *rpmA*, *rpmC*, *rpmI*, *rpoA*, *rpoB*, *rpoC*, *rpsB*, *rpsC*, *rpsD*, *rpsE*, *rpsF*, *rpsG*, *rpsH*, *rpsI*, *rpsJ*, *rpsK*, *rpsL*, *rpsM*, *rpsO*, *rpsP*, *rpsQ*, *rpsR*, *rpsS*, *rpsT*, *secA*, *secG*, *secY*, *serS*, *smpB*, *tig*, *tilS*, *truB*, *tsaD*, *tsf*, *uvrB*, *ybeY*, and *ychF*.

The final genome assembly of TJS119 contained nine contigs with a total length of 3,809,913 bp, an N50 of 2,061,305 bp, 151 × sequencing depth, and 3,750 protein-coding regions. Genome sequencing indicated that the DNA G + C content of TJS119 was 46.4 mol% (Table 1). The chromosome contains 73 tRNAs, 10 rRNAs, and 1 tmRNA. The ANI between TJS119 and *B. velezensis* was 98.26. The phylogenomic tree (Figure 3), based on 92 core bacterial gene sequences, also indicated that TJS119 is a *B. velezensis* strain. The genomic features of TJS119 were compared with those of other closely related *Bacillus* spp. as shown in Table 2. The genome size of the five *B. velezensis* strains ranged from 3.80 to 3.92 Mb; their G + C content ranged from 46.4 to 46.5% and predicted number of coding genes ranged from 3,684 to 3,750. TJS119 and five other strains possesses one circular chromosome without a plasmid.

**Table 1:** Genome features of *Bacillus velezensis* TJS119.

Attribute	Value
Genome size (bp)	3,809,913
No. of contigs	9
G + C content	46.46 mol%
Total genes	3,834
N50	2,061,305
Sequencing depth	151x
tRNA genes	73
rRNA genes	10
Protein-coding genes	3,750

**Table 2:** Comparison of genomic features between *Bacillus velezensis* TJS119 and closely-related strains.

Features	*B. velezensis* TJS119	*B. velezensis* VR-34	*B. velezensis* HBXN2020	*B. velezensis* JB7	*B. velezensis* JB8	*B. amyloliquefaciens* DSM7
Genome size (bp)	3,809,913	3,891,665	3,929,792	3,929,792	3,929,735	3,980,199
G + C content (%)	46.4	46.5	46.5	46.5	46.5	46.1
Protein-coding genes	3,750	3,686	3,684	3,685	3,689	4.135
tRNA	73	80	86	86	86	24
rRNA	10	15	27	27	27	30

The ANI is a powerful approach for assessing evolutionary distances among bacterial species based on digital whole-genome comparisons, and the closer the value to 1 the higher the similarity. Based on the ANI values, the genome sequence of TJS119 is most similar to that of *B. velezensis* with ANI values > 97%. However, the ANI values between TJS119 and *B. amyloliquefaciens* were < 95%. Strains with ANI > 96% are typically considered to belong to the same species. Therefore, TJS119 does not belong to *B. amyloliquefaciens*.

The antiSMASH 7.0 webserver revealed 12 BGCs in the genome of *B. velezensis* TJS119, eight of which presented significant similarity with previously identified clusters in the Minimum Information about a Biosynthetic Gene cluster (MIBiG) repository that are involved in the synthesis of surfactin (82% similarity to known clusters revealed by antiSMASH), macrolactin H (100%), bacillaene (100%), fengycin (100%), difficidin (100%), bacillibactin (100%), bacilysin (100%), and butirosin A/butirosin B (7%). Four of the 12 clusters were not similar to any of the clusters in the antiSMASH database (Table 3). We compared the genome sequence of *B. velezensis* TJS119 with sequences on the VFDB database. Only a few virulence genes related to adherence, immune evasion, iron acquisition, regulation, secretion system, toxin, acid resistance, antiphagocytosis, cell surface components, copper uptake, invasion, iron uptake, stress adaptation, and surface protein anchoring factor were detected in the genome data of *B. velezensis* TJS119 (Supplementary Table S1). No pathogenic genes were detected in the genome.

**Table 3:** Biosynthetic gene clusters (BGCs) found in the TJS119 genome using the webserver antiSMASH 7.0.0.

Contig	Cluster	Type	From (pb)	To(bp)	Most similar known cluster		Similarity
1	1	transAT-PKS	546,636	638,995	Difficidin	Polyketide	100
	2	T3PKS	767,630	808,356			
	3	Terpene	873,696	893,822			
	4	NRPS, betalactone, and transAT-PKS	922,559	1,056,869	Fengycin	NRP	100
	5	transAT-PKS, NRPS, and T3PKS	1,130,611	1,231,176	Bacillaene	polyketide +NRP	100
	6	transAT-PKS	1,450,364	1,538,597	Macrolactin H	Polyketide	100
	7	Lanthipeptide-class-ii	1,705,160	1,734,048			
	8	terpene	1,854,541	1,875,281			
	9	PKS-like	1,957,325	1,998,569	Butirosin A, B	Saccharide	7
2	10	NRP-metallophore, NRPS, and RiPP-like	73,687	125,478	Bacillibactin	NRP	100
	11	Other	661,788	703,206	Bacilysin	Other	100
3	12	NRPS	199,693	265,100	Surfactin	NRP: Lipopeptide	82

NRPS, Non-ribosomal peptide synthetase; NRP, Non-ribosomal peptide; PKS, Polyketide synthetase; AT, Acetyltransferase; and T3PKS, Type 3 Pks.

### 3.3 *In vitro* antifungal activity

**Figure 4:**
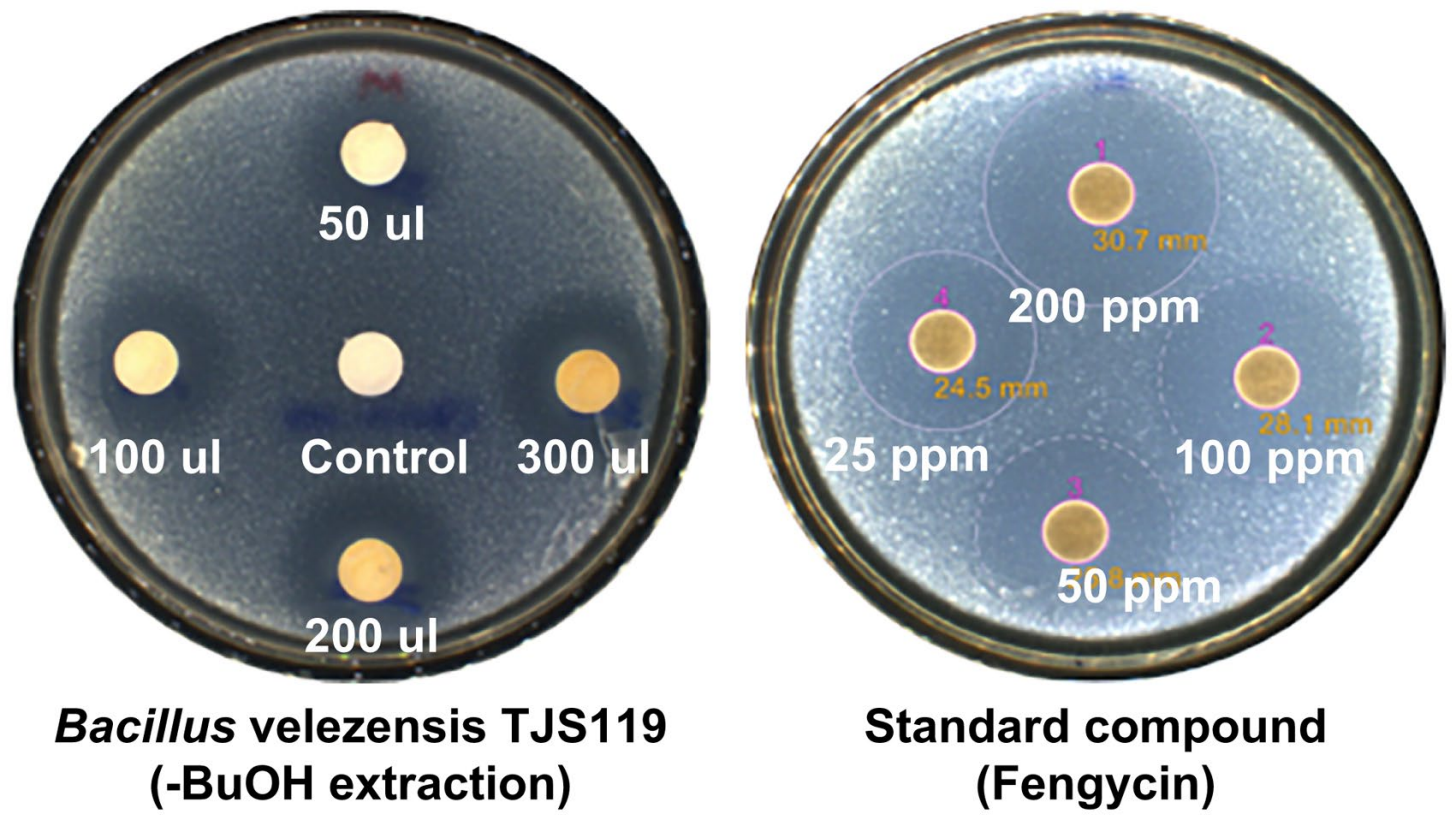
Antifungal activity of *Bacillus velezensis* TJS119 against *Metarhizium anisopliae.*

The butanol extract of TJS119 culture broth inhibited the growth of *M. anisopliae* in a concentration-dependent manner (Figure 4). This result confirmed the presence of antifungal compounds in the n-butanol extract. The fengycin standard (Sigma-Aldrich) inhibited *M. anisopliae* at all concentrations tested (25–200 ppm).

### 3.4 Identification of antifungal compounds

**Figure 5:**
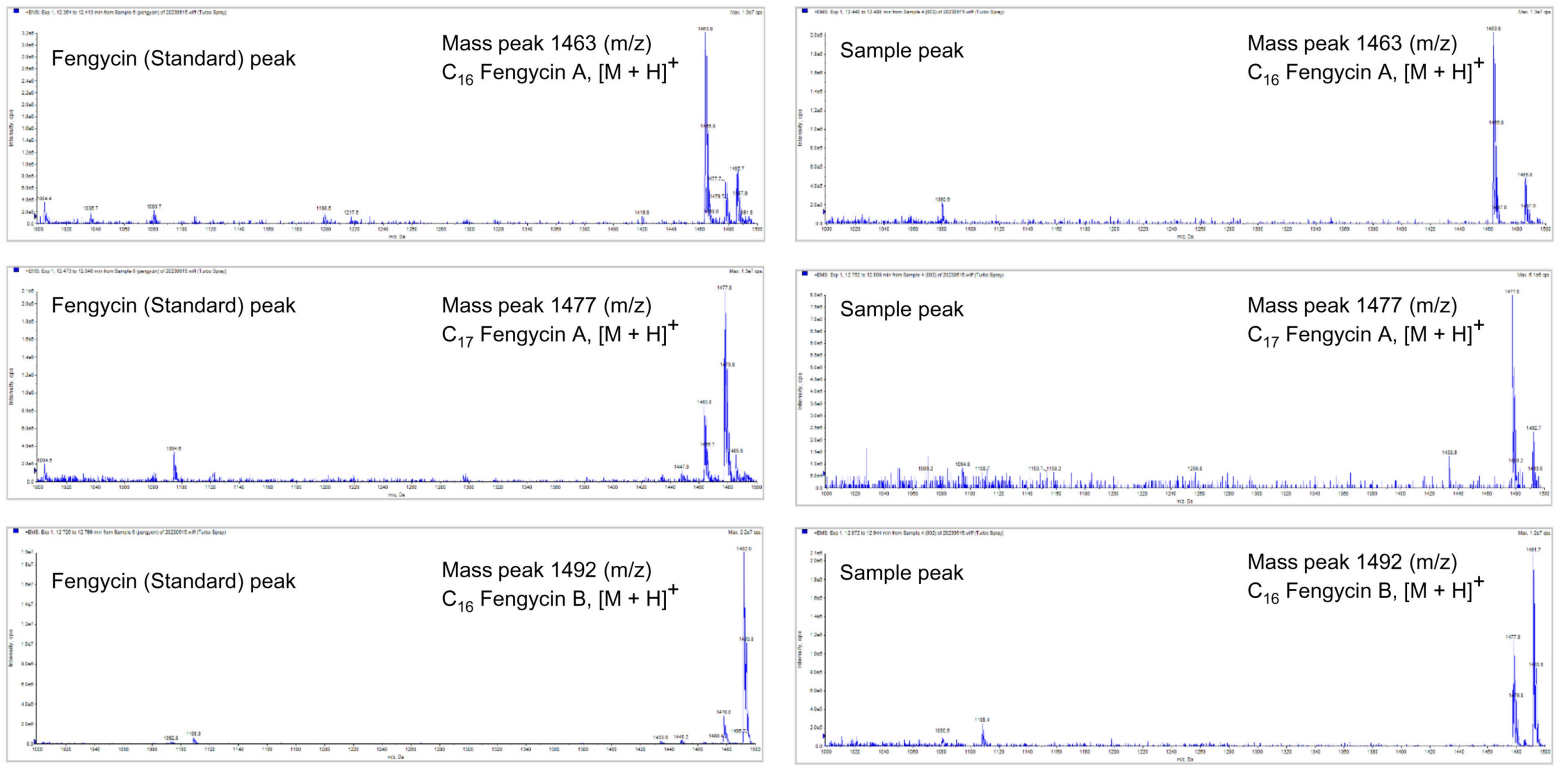
LC–MS/MS-based fragmentation analysis of fengycin (m/z 1463.8, 1477.7, and 1491.8) isolated from the n-butanol extract of *Bacillus velezensis* TJS119.

To identify the antifungal compounds produced by the bacterial isolate, the extract of TJS119 was subjected to UPLC–MS/MS analysis. The molecular ion masses of previously reported compounds were obtained from their LC–MS analysis. The UPLC–MS/MS data were analyzed for the presence of fingerprints of the compounds by matching them to those of previously reported structures. The results of the UPLC-MS/MS analysis suggested the presence of fengycin [m/z 1,063.8, 1,477.7, 1,491.8 (M + H^+^); Figure 5; Table 4].

**Table 4:** Summary of the masses of compounds identified using LC-MS analysis.

No.	Mass peak (*m*/*z*)	Positive ionization (M + H^+^)	Compound identified with reported mass
1	1463.8	C_16_ Fengycin A, [M + H]^+^	Fengycin class
2	1477.7	C_17_ Fengycin A, [M + H]^+^	
3	1491.8	C_16_ Fengycin B, [M + H]^+^	

### 3.5 Biocontrol effect of TJS119 against *Metarhizium anisopliae*

One week 5, the incidence rate of fungal infection in the group was 60.83% ± 4.12%; whereas the incidence rate was 3.17% ± 4.08% when treated with *B. velezensis* TJS119 (1 × 10^7^ CFU/g). The biocontrol effect of TJS119 against green muscardine disease (*M. anisopliae*) was 94.7%. The bioassay results indicated that TJS119 could control green muscardine disease (Table 5).

**Table 5:** Evaluation of biocontrol efficacy of *Bacillus velezensis* TJS119 against green muscardine disease.

	Disease index	Biocontrol effect
Control	60.83 ± 4.12	-
TJS119	3.17 ± 4.08	94.70%

## 4 Discussion

The genus *Bacillus* includes several species that are highly similar, and the taxonomic status of *Bacillus* strains cannot be easily determined using conventional classification methods (Hamdache et al., 2013). Several strains initially identified as *B. amyloliquefaciens*, including FZB42 (Schilling et al., 2018), vb7 (Saravanan et al., 2021), and SQR9 (Feng et al., 2018), are now classified as *B. velezensis*. To determine the relationship between TJS119 and other strains of *Bacillus*, a phylogenetic tree based on 92 core gene sequences was constructed in this study. The results indicate that TJS119 is closely related to *B. velezensis* NRRL B-41580^T^, *B. amyloliquefaciens* DSM 7 ^T^, and *B. siamensis* KCTC 13613^T^.

Among biocontrol bacteria, *Bacillus* spp. are the most effective. The primary mechanisms employed by *Bacillus* spp. in disease control in plants involve antagonism, competition, and disease resistance induction. In addition, *Bacillus* spp. can enhance resistance by promoting plant growth in a synergistic manner (Backer et al., 2018; Rodriguez et al., 2019). However, no study has evaluated their ability to prevent diseases in industrial insect husbandry.

Recent studies have focused on secondary metabolites and enzymes produced by *B. velezensis* that confer resistance in plants against a broad-spectrum of pathogens (Wang et al., 2021). Antimicrobial cyclic lipopeptides synthesized by NRPS and polyketides synthesized via the polyketide pathway have broad-spectrum antimicrobial activity. Cyclic lipopeptides belong to three families, surfactin, iturin, and fengycin, all of which can induce systemic resistance in plants against pathogens (Wu et al., 2018). However, the gene cluster encoding iturin was not detected in TJS119. Polyketides, including macrolactins, bacillysins, and difficidins, can be used as antibiotics, antifungals, and natural pesticides. The most important *Bacillus* polyketides are those of *B. subtilis* and *B. velezensis*, with the former being particularly rich in these compounds (Rabbee and Baek, 2020). Using whole-genome sequencing, Zhang et al. (2022) found that *B. velezensis* GS-1 produces the lipopeptides surfactin, fengycin, and plantazolicin, which exhibit inhibitory effects against *M. oryzae*. Eight secondary metabolite-related gene clusters have been identified in *B. velezensis* VB7, which is protective against carnation infection by *Sclerotinia sclerotiorum*, cotton infection by tobacco streak virus, and tomato peanut shoot necrosis infection (Saravanan et al., 2021). Meanwhile, 12 secondary metabolite-related gene clusters have been identified in *B. velezensis* YC89, which effectively controls red rot disease in sugarcane (Xie et al., 2023). In the present study, 12 gene clusters were predicted in the TJS119 genome using antiSMASH, an online secondary metabolite gene cluster prediction tool, including four with unknown functions and seven highly similar antibiotic synthesis gene clusters (serpactin, macrolactin H, bacillin, fengycin, dipicidin, bacillibactin, and bacillicin). The 12 secondary metabolite gene clusters were identified using whole-genome sequencing. We further confirmed that fengycin was the main active secondary metabolite in the organic solvent extract.

The safety of bacteria used for biological control was confirmed. The analysis of genome of *B. velezensis* TJS119 against the VFDB database revealed the presence of some putative virulence genes, which cannot be considered harmful. Genes encoding hemolysin A (hlyA), cytolysin (cyl), enterotoxins hemolysin BL (Hbl), non-hemolytic enterotoxin (Nhe), and cytotoxin K (CytK), which are well-known potential virulence factors, are missing in *B. velezensis* TJS119 (Dietrich et al., 2021; Chen et al., 2023). In addition, no truly pathogenic coding genes were found in the genome of *B. velezensis* TJS119. Additionally, studies on *B. velezensis* using fish (Gao et al., 2017), pig (Bampidis et al., 2021), layer chicken (Ye et al., 2020), and mice (Chen et al., 2023) have suggested the safety of this strain controlling diseased in animals.

In the present study, we assessed the relative efficacy of *B. velezensis* TJS119 against green muscardine disease using laboratory tests. *Bacillus velezensis* TJS119 exhibited a relative efficacy of 94.7% against green muscardine disease. The successful introduction of biocontrol agents can be influenced by environmental various factors in the field, such as temperature, climate, and terrain. Therefore, further studies, particularly field trials with *B. velezensis* TJS119, are necessary.

## 5 Conclusion

In the field of industrial insect husbandry, the breeding of *P. brevitarsis seulensis* larvae is challenged by substantial mass mortality, primarily linked to the onset of diseases, specifically green muscardine disease. The use of chemical fertilizers and antifungal agents to increase production is unsustainable owing to their conspicuous environmental effects. Based on the findings of this study, *B. velezensis* TJS119 can be considered an alternative biocontrol agent to be formulated and extensively applied in bio-based industries. Our *in vitro* experiments and *in vivo* bioassay provide information on the genomic features of *B. velezensis* TJS119. The identified secondary metabolite clusters and the comparative analysis of the implicated genes indicate that the biological control properties of *B. velezensis* TJS119 are genetically associated, and thus inherently stable.

## Data availability statement

The datasets presented in this study can be found in online repositories. The names of the repository/repositories and accession number(s) can be found at: https://www.ncbi.nlm.nih.gov/genbank/, JAXQPU000000000.

## Author contributions

K-IH: Conceptualization, Methodology, Project administration, Visualization, Writing – original draft, Writing – review & editing. YN: Investigation, Resources, Writing – review & editing. BH: Formal Analysis, Investigation, Writing – review & editing. JK: Investigation, Writing – review & editing. JJ: Investigation, Resources, Writing – review & editing. EK: Funding acquisition, Writing – review & editing. M-HL: Supervision, Writing – review & editing.

## Footnotes

^1^ https://www.ezbiocloud.net/tools/orthoani

^2^ https://antismash.secondarymetabolites.org/

^3^ https://www.ncbi.nlm.nih.gov/

^4^ https://mibig.secondarymetabolites.org/
